# Association between Serum Interleukin-6 Concentration and Mortality in Patients with Coronary Artery Disease

**DOI:** 10.1155/2013/726178

**Published:** 2013-05-30

**Authors:** Dongfang Su, Zhongxia Li, Xinrui Li, Yuming Chen, Yuan Zhang, Ding Ding, Xueqing Deng, Min Xia, Jian Qiu, Wenhua Ling

**Affiliations:** ^1^Guangdong Provincial Key Laboratory of Food, Nutrition, and Health, Department of Nutrition, School of Public Health, Sun Yat-Sen University, 74 Zhongshan Road 2, Guangzhou, Guangdong 510080, China; ^2^Department of Cardiology, Guangzhou Military General Hospital, Number 111 Liuhua Road, Guangzhou, Guangdong 510010, China; ^3^Pennington Biomedical Research Center, Baton Rouge, LA 70808, USA

## Abstract

*Objectives*. To evaluate whether serum interleukin-6 (IL-6) is associated with increased risk of mortality in coronary artery disease (CAD) patients. *Methods*. We performed a prospective cohort study of 718 CAD patients from the Guangzhou Cardiovascular Disease Cohort (GCDC) study. Multivariable-adjusted Cox proportional hazards regression analyses were used to examine the association between serum IL-6 with all-cause and cardiovascular mortality. *Results*. During the 1663 person-years of followup, the cumulative all-cause mortality and cardiovascular mortality were 6.5% (*n* = 47) and 3.3% (*n* = 24), respectively. The mean length of followup was 2.32 ± 0.81 years. In the multivariable analyses, a one-SD increment in log-transformed serum IL-6 was positively associated with an increased risk of all-cause and cardiovascular mortality, with hazard ratios (HR) of 2.93 (95% CI, 2.11–4.08) and 2.04 (95% CI, 1.34–3.68) within the patients combined and 2.98 (95% CI, 2.12–4.18) and 3.10 (95% CI, 1.98–4.85) within males, respectively. Patients in the highest serum IL-6 tertile versus the lowest tertile were at higher risk of all-cause and cardiovascular mortality, with HR of 17.12 (95% CI 3.11–71.76) and 8.68 (95% CI, 1.88–37.51), respectively. *Conclusions*. In hospitalized patients with CAD, serum IL-6 is significantly associated with all-cause and cardiovascular mortality.

## 1. Introduction

Inflammatory biomarkers have been shown to be associated with and to predict the onset of cardiovascular events [1–3]. The predictive value of these markers, including interleukin-6 (IL-6), has been demonstrated for subjects with existing coronary artery disease (CAD) and apparently healthy subjects [3–6].

There is an extensive body of the literature that supports the role of chronic inflammation in the development and progression of atherosclerosis [7]. It has been reported that increased levels of inflammatory agents, including IL-6, are associated with acute ischemic conditions and are predictors of recurrent events in patients with CAD [1, 2, 7]. The serum level of IL-6, along with other cytokines, is also associated with unfavourable clinical outcomes in patients hospitalised for unstable angina and ST-elevated myocardial infarction (STEMI) [6, 8, 9]. Furthermore, many chronic conditions that are common causes of death in older persons may stimulate and sustain a systemic inflammatory state, which can be measured by increased levels of serum IL-6 or other proinflammatory cytokines. The secretion of IL-6, which is a major determinant of the production of acute-phase proteins, is increased in clinical situations characterised by tissue injury, including infections, malignant neoplasms, ischemic diseases, and trauma [10]. This pathophysiology may also explain the elevated risk of mortality associated with increased circulating levels of inflammatory markers.

Only a few population-based studies have investigated the association between circulating levels of serum IL-6 and the risk of all-cause and cardiovascular mortality in CAD patients [6–8]. The Guangzhou Cardiovascular Disease Cohort (GCDC) study is an ongoing multicentre, prospective observation study of the risk factors of cardiovascular disease (CVD) that has generated detailed information. This dataset gave us the opportunity to investigate the value of IL-6 as an independent predictor of the 2.3-year risk of mortality in CAD patients.

## 2. Materials and Methods

### 2.1. Study Population

The details of the cohort, selection, criteria, and purpose of the GCDC study have been published elsewhere [11–13]. Briefly, between 2008 and 2012, about 3500 patients aged between 40 and 80 years were selected from four hospitals. Patients were eligible if they were diagnosed with CAD, hypertension, diabetes mellitus (DM), or stroke by a cardiologist according to the criteria of the American Heart Association Council on Epidemiology and Prevention [14]. As part of the GCDC study, this analysis covered 1050 CAD patients. The inclusion criteria were significant CAD defined as a greater than 20% stenosis in at least one major coronary artery [15]. Of the 1050 patients, 250 did not have a baseline IL-6 measurement and 72 patients had no follow-up information and so were excluded. We also excluded 10 patients who died within three months of blood time, resulting in a final sample of 718 participants. The study was approved by the Institutional Review Board of the Sun Yat-sen University Health Science Centre. All of the participants provided written informed consent.

### 2.2. Data Collection

A face-to-face interview was performed with each participant by a trained postgraduate or medical undergraduate from Sun Yat-sen University. The data on general information of examination date, sex, birth date and place, address, occupation, education level, leisure time physical activity, smoking habits, alcohol and tea consumption, family history of CAD, medication history, and a validated food frequency questionnaire [16] were conducted as previously published [11–13]. Smoking was defined as at least one cigarette a day and lasting more than half a year. Smoking status was classified as never, past, or current. Alcohol drinking was defined as persons who drank any type of alcoholic beverage at least once a week [17] and lasted more than six months.

### 2.3. Biochemical Measures

Peripheral venous blood was obtained the next day after the patients were diagnosed with CAD following an overnight fast and centrifuged at 4°C and 3360 g for 15 min. All of the samples were then stored at −80°C until analysis. The total cholesterol (TC), triglyceride (TG), and high-density lipoprotein cholesterol (HDL-c) concentrations were determined enzymatically. The low-density lipoprotein cholesterol (LDL-c) level was assayed using an indirect method. We measured the serum levels of IL-6 and CRP with the Human Basic Kit FlowCytomix (BMS8420FF, eBioscience, USA) and the Human FlowCytomix (Simplex BMS8213FF and BMS8288FF, eBioscience, USA) on a BD FACSCalibur instrument (BD Biosciences, USA) according to the manufacturer's instructions. Data were obtained using the CellQuest software (BD Biosciences) and calculated using the FlowCytomix Program (eBioscience, USA). The low detection limit of the assay was 1.01 pg/mL and 0.1 ng/mL for IL-6 and CRP, respectively. The mean inter-assay and intra-assay coefficients of variation were 7.1%, 9.9% and 6.2%, 5.6%, respectively, for IL-6 and CRP.

### 2.4. Outcomes and Quality Control

The primary outcome of the study is all-cause mortality, which includes cardiovascular mortality and death due to other causes (accident, cancer, liver or kidney failure, and injury). Cardiovascular death includes sudden death and death caused by pump failure, acute myocardial infarction (AMI), stroke, and heart failure (HF) or after a cardiovascular procedure. Semiannual telephone interviews were conducted with patients or their proxy to ask about death or hospitalisation. When a suspicious cardiovascular event recurrence was reported, we accessed the hospital's medical records, death certificates, or forensic inspection reports and confirmed whether a particular event actually occurred with a committee of cardiologists. All clinical events were defined according to the American Heart Association Council on Epidemiology and Prevention [14].

### 2.5. Statistical Methods

Descriptive statistics were used to compare the baseline characteristics across quartiles of log-transformed IL-6 levels. A chi-square test and one-way ANOVA were used for the categorical and continuous variables and a Mann-Whitney *U* or Kruskal-Wallis *H* test was used for variables that were not normally distributed.

Cox proportional hazards models were used to examine the hazard ratio (HR) of all-cause and cardiovascular mortality associated with serum IL-6 and CRP levels as continuous variables (log transformed) and also divided into tertiles as categorical variables (by gender). The adjusted confounders were as follows: model 1: age, sex, and body mass index (BMI); model 2: model 1 plus smoking, alcohol use, hypertension, DM, LDL-c, and HDL-c; model 3: model 2 plus CRP or IL-6. We stratified our analysis by males only, because only eight of the females died during followup, with one cardiovascular death.

We used the Kaplan-Meier method analysis to compare the survival curves for serum IL-6 levels with all-cause and cardiovascular mortality. The cumulative survival rates of patients with different serum IL-6 levels were compared using a log-rank test.

The statistical significance was set as *P* < 0.05. All of the computations were carried out with SPSS 16.0 software (SPSS, Inc., Chicago, Illinois).

## 3. Results and Discussion

### 3.1. Description of the Population

The baseline demographic, clinical and laboratory characteristics of the 718 patients (63% male) are shown in Table 1. The mean age (±standard deviation, SD) and BMI of the subjects were 64 (±11) years and 23.9 (±3.6) kg/m^2^, respectively. Patients with higher serum IL-6 levels (quartile 4) were older, more likely to be DM, current smokers, and to be separated, and less likely to be married than patients with low IL-6 levels (quartile 1). Additionally, patients with higher serum IL-6 levels in quartiles 2 to 4 had higher CRP levels of 2.16 (0.55–8.38), 3.39 (1.01–12.07) and 6.37 (2.89–16.90) ug/mL, respectively, (all *P* values < 0.001) than patients with low serum IL-6 levels. Patients with low IL-6 levels (quartile 1) had higher HDL-c levels than patients with high IL-6 levels (1.13 versus 1.06 mmol/L, *P* = 0.024).

### 3.2. Mortality during Followup

During the 1663 person-years of followup, the all-cause mortality and cardiovascular mortality of patients with serum IL-6 levels in tertiles 1 to 3 were 3.7, 15.1, and 68.8 and 3.7, 5.0, and 36.3 per 1000 person-years, respectively (Table 2). The mean length of followup was 2.32 ± 0.81 years.

### 3.3. Hazard Ratios for All-Cause and Cardiovascular Mortality

For each additional SD, even after adjustment for age, sex, BMI, smoking, alcohol use, hypertension, DM, LDL-c, HDL-c, and CRP, the log-transformed serum IL-6 levels were positively associated with a higher risk of all-cause and cardiovascular mortality, with hazard ratios of 2.93 (95% CI, 2.11–4.08, *P* < 0.001) and 2.04 (95% CI, 1.34–3.68, *P* < 0.001), respectively (see Table 2). When stratified into tertiles, higher serum IL-6 levels (tertile 3) were significantly associated with both all-cause mortality (HR: 17.12, 95% CI, 3.11–71.76, *P* < 0.001) and cardiovascular mortality (HR: 8.68, 95% CI, 1.88–37.51, *P* < 0.001) compared with the reference group even after adjustment for potential confounders in model 3 (see Table 2).

**Figure 1:**
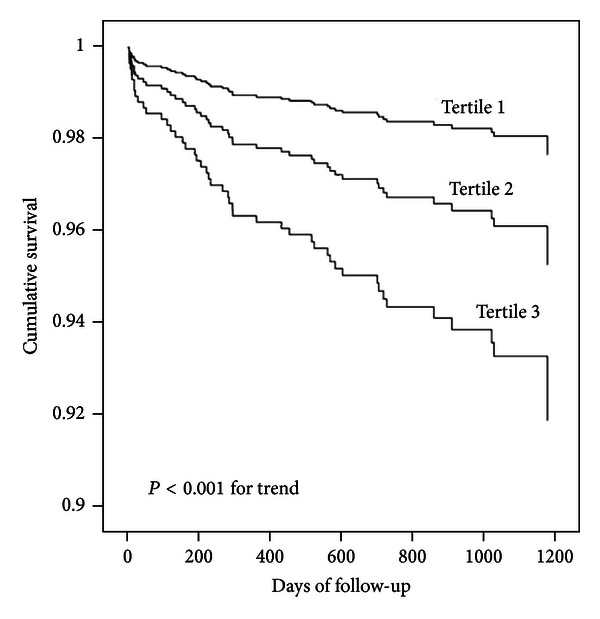
Kaplan-Meier hazard plots for all-cause mortality by tertiles of interleukin-6. The probability values represent the trend across all three tertiles.

**Figure 2:**
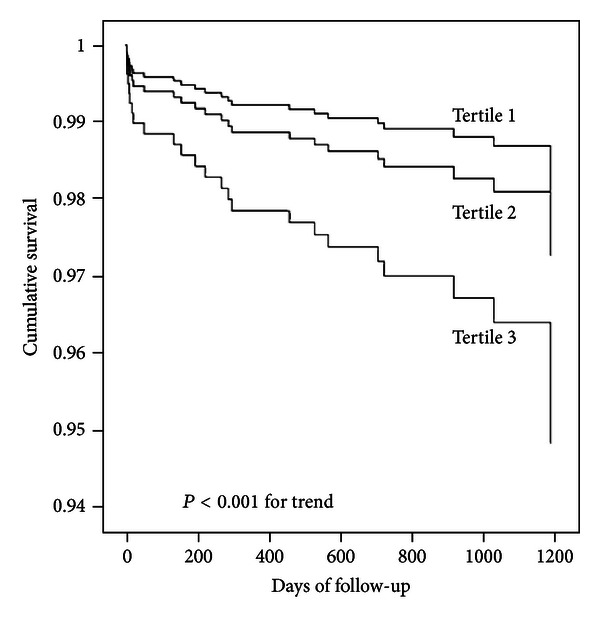
Kaplan-Meier hazard plots for cardiovascular disease mortality by tertiles of interleukin-6. The probability values represent the trend across all three tertiles.

In stratified analysis in males, for each additional SD, even after adjustment for potential confounders in Table 2, the log-transformed serum IL-6 levels were positively associated with a higher risk of all-cause and cardiovascular mortality, with hazard ratios of 2.98 (95% CI, 2.12–4.18, *P* < 0.001) and 3.10 (95% CI, 1.98–4.85, *P* < 0.001), respectively (see Table 3). When stratified into tertiles, higher serum IL-6 levels (tertile 3) were significantly associated with both all-cause mortality (HR: 11.68, 95% CI, 3.04–37.56, *P* < 0.001) and cardiovascular mortality (HR: 8.37, 95% CI, 1.93–36.22, *P* < 0.001) compared with the reference group even after adjustment for potential confounders in model 3 (see Table 3, Figures 1 and 2).

### 3.4. Main Findings

In these hospitalized CAD patients with a mean followup of 2.32 years, increased serum IL-6 levels were associated with an increased risk of all-cause and cardiovascular mortality even after adjusted for potential confounders, suggesting a possible pathophysiological role of this proinflammatory cytokine in the process leading to death.

### 3.5. Association of IL-6 and Mortality in the Published Literature

Several previous studies have probed the association between serum IL-6 and mortality, with conflicting results. The Women's Health and Aging study showed that among women with prevalent CVD, those with higher plasma IL-6 levels had a more than fourfold risk of death (RR: 4.6, 95% CI, 2.0–10.5) compared with women in the lowest tertile, but the study did not find this association among those without CVD [18]. In contrast, Tuomisto et al. reported that CRP and TNF-*α* but not IL-6 were significant independent predictors of total mortality among men [19]. Arai reported that serum IL-6 was not associated with all-cause mortality in 285 subjects with a mean age of 101.5 [20]. However, Scharnagl et al. found IL-6 to be more strongly associated with all-cause and cardiovascular mortality than CRP [5]. Other recent studies have shown that elevated levels of serum IL-6 provide valuable information for the risk assessment of long-term cardiovascular mortality in patients with STEMI and are a powerful predictor of cardiovascular and all-cause mortality [8, 21]. These conflicting findings may be explained by the small sample size in several of the studies, the old age of the participants, and heterogeneous populations. Our hospitalised cohort study enrolled a larger number of CAD patients with a homogeneous status who were younger than those in some of the aforementioned studies. Our findings show a clear association between serum IL-6 and all-cause and cardiovascular mortality in existing CAD patients.

Few studies have examined the association between IL-6 and mortality in Chinese CAD patients. A recent study found that plasma IL-6 level predicts short- and long-term mortality in patients with acute heart failure (HF) [4]. In two prospective studies, long-term serum IL-6 levels were associated with CHD [22]. Haugen et al. showed that an increased IL-6 concentration predicts mortality in elderly HF patients [23]. Panichi et al. reported plasma IL-6 to be a stronger predictor of total and cardiovascular mortality than CRP in haemodialysis patients [24]. Our study confirms these reports of positive associations between circulating IL-6 concentration and subsequent risk of mortality, finding that serum IL-6 is a stronger predictor of total and cardiovascular mortality than CRP, which supports the potential role of inflammation in the progression and prognosis of CAD. Additionally, in our stratified analysis, serum IL-6 was positively associated with increased risk of all-cause and cardiovascular mortality in males even after adjustment for potential confounders. There were only 8 females among the 47 patients who died, and thus, the data were insufficient for a detailed analysis of the female patients, which is a limitation of the study. However, this situation may change as the followup extends. The issue of gender-specific aspects of mortality, including inflammatory markers, is still controversial [25]. It is thus necessary to further study the gender specificity of mortality in relation to the inflammatory hypothesis and CAD.

### 3.6. Potential Mechanisms

The proinflammatory cytokine IL-6 has been extensively studied. The key mechanisms by which circulating IL-6 contributes to the development of CAD are summarised elsewhere [26]. First, serum IL-6 is the main stimulator of hepatic acute-phase response, which is associated with increased blood viscosity and increased platelet number and activity. Second, the autocrine and paracrine activation of monocytes by IL-6 in the vessel wall contributes to the deposition of fibrinogen [27]. Acute-phase response proteins such as CRP and fibrinogen are both strong risk factors for CAD. Third, IL-6 decreases the activity of lipoprotein lipase (LPL) and the levels of monomeric LPL in plasma, thereby increasing the uptake of lipids by macrophages [28]. Fourth, circulating IL-6 also stimulates the hypothalamic-pituitary-adrenal (HPA) axis, the activation of which is associated with central obesity, hypertension, and insulin resistance [29].

A large number of studies report a positive association between serum IL-6 concentration and the risk of mortality from CAD [1, 6, 30–32]. However, whether elevated serum IL-6 plays a causal role in CAD mortality remains unclear. A recent report showed that IL-6 may play a causal role in the development of coronary heart disease [33] as interleukin-6 receptor (IL6R) blockade reduced systemic and articular inflammation. Furthermore, this causal association between IL6R-related pathways and coronary heart disease is also strongly supported by a collaborative meta-analysis [34]. These results suggest that targeting IL6R could provide a novel therapeutic approach to the prevention of coronary heart disease. Our study found that serum IL-6 levels were significantly higher at baseline in patients who died during followup, in line with previous studies, thus supporting the notion that the chronic inflammatory process may play a causal role in the development and prognosis of atherosclerotic disease. Further research is needed to confirm the causality of association between serum IL-6 levels and mortality.

### 3.7. Limitations and Strengths of Our Study

To the best of our knowledge, this is the first study to show that serum IL-6 is associated with all-cause and cardiovascular mortality in a hospitalised cohort of Chinese CAD patients. However, although the study has a prospective design, the findings should be interpreted with caution, in that cytokine IL-6 is not to be regarded as a causative factor of death but rather a possible long-term biomarker of mortality in CAD patients. Also, the study samples and incident cases are small, and further investigations in a large number of subjects are needed to confirm the present findings. Although in this study cytokine measures were employed only at baseline, other studies suggest that prospective changes in inflammatory markers are better predictors of mortality than baseline measures [35]. However, a more recent systematic review reported that even ignoring the variability, increasing IL-6 levels are still associated with progressively increasing CHD risk, and the 17 available prospective studies gave a combined odds ratio of 1.61 (95% CI, 1.42–1.83) per 2 SD increase in baseline IL-6 (corresponding to an odds ratio of 3.34 (95% CI, 2.45–4.56) per 2 SD increase in the usual (long-term average) IL-6 levels). Our findings thus have some merit, although they require replication in studies with multiple time points of inflammatory measures. We considered diseases associated with inflammation, but were unable to consider other potential factors associated with inflammation such as diet. However, the homogeneous status of the GCDC participants as ethic Han Chinese from the same area with similar dietary patterns means that diet may not have had a significant effect on the study findings.

## 4. Conclusions

In Chinese hospitalised patients with CAD, serum IL-6 concentrations were associated with all-cause and cardiovascular mortality independent of potential confounders. This medium-scale prospective analysis provides reliable evidence of the role of serum interleukin-6 in the progression and prognosis of atherosclerotic disease. Our findings are consistent with and extend those of previous studies showing the potential of inflammatory pathways as targets for cardiovascular disease prevention and highlight the need for studies of IL-6 signalling inhibition for the prevention of coronary artery disease.

## Figures and Tables

**Table 1:** Baseline clinical characteristics according to total and quartiles of serum interleukin-6.

	Total	Quartile 1	Quartile 2	Quartile 3	Quartile 4	*P* trend	P*
Range, pg/mL	0.10–101.63	≤1.31	1.32–2.39	2.40–4.26	4.27–101.63	—	—
No. of patients	718	180	179	180	179	—	—
Time of followup, y	2.32 ± 0.81	2.26 ± 0.63	2.47 ± 0.72	2.37 ± 0.86	2.19 ± 0.80	**0.006**	0.429
Age, y	64 ± 11	63 ± 11	64 ± 11	63 ± 11	65 ± 11	0.114	**0.022**
Male sex (*n*, %)	454 (63)	108 (59)	113 (62)	111 (60)	122 (67)	0.431	0.114
Hypertension (*n*, %)	673 (93)	167 (91)	167 (91)	170 (92)	169 (92)	0.924	0.708
Diabetes mellitus (*n*, %)	158 (22)	25 (14)	40 (22)	43 (23)	50 (27)	**0.012**	**0.001**
Current smoker (*n*, %)	200 (28)	38 (21)	58 (32)	50 (28)	54 (30)	0.094	**0.032**
Current drinker (*n*, %)	79 (11)	21 (12)	21 (12)	15 ( 8)	22 (12)	0.626	0.500
Education							
Middle school and below (*n*, %)	344 (48)	90 (50)	81 (45)	79 (44)	94 (53)	0.320	0.355
High school (*n*, %)	195 (27)	46 (26)	50 (28)	46 (26)	53 (30)	0.785	0.229
Above high school (*n*, %)	170 (24)	42 (23)	45 (25)	53 (29)	30 (17)	0.038	0.073
Missing (*n*, %)	9 (1)	2 (1)	3 (2)	2 (1)	2 (1)	0.952	0.686
Marriage							
Single (*n*, %)	119 (17)	19 (11)	30 (17)	40 (22)	30 (17)	**0.031**	0.059
Married (*n*, %)	447 (62)	131 (73)	111 (62)	101 (56)	104 (58)	**0.005**	**0.002**
Separated (*n*, %)	138 (19)	27 (15)	33 (18)	37 (21)	41 (23)	0.272	**0.038**
Missing (*n*, %)	14 (2)	3 (2)	5 (3)	2 (1)	4 (2)	0.687	0.497
Body mass index, kg/m^2^	23.91 ± 3.58	23.92 ± 4.03	24.07 ± 3.18	23.71 ± 3.50	23.92 ± 3.60	0.822	0.985
Total cholesterol (mmol/L)	4.75 ± 1.13	4.74 ± 1.16	4.77 ± 1.06	4.85 ± 1.22	4.63 ± 1.06	0.290	0.334
Triglyceride (mmol/L)	1.82 ± 1.26	1.72 ± 1.00	2.07 ± 1.76	1.79 ± 1.17	1.69 ± 0.92	**0.018**	0.812
LDL-c (mmol/L)	3.01 ± 0.97	3.03 ± 0.97	2.95 ± 0.91	3.10 ± 1.05	2.94 ± 0.94	0.313	0.358
HDL-c (mmol/L)	1.09 ± 0.30	1.13 ± 0.30	1.08 ± 0.28	1.06 ± 0.35	1.06 ± 0.29	0.097	**0.024**
C-reactive protein	3.15 (0.74–10.27)	1.21 (0.33–5.11)	2.16 (0.55–8.38)	3.39 (1.01–12.07)	6.37 (2.89–16.90)	**<0.0001**	**<0.0001**
Interleukin-6	2.39 (1.31–4.26)	0.85 (0.56–1.11)	1.82 (1.55–2.07)	3.25 (2.77–3.74)	6.93 (5.36–11.42)	**<0.0001**	**<0.0001**

The values are the mean ± SD for the continuous variable if normally distributed, the median (25th, 75th) for nonnormally distributed variables, and *n* (%) for the categorical variables. *Quartile 4 versus quartile 1. LDL-c: low-density lipoprotein cholesterol; HDL-c: high-density lipoprotein cholesterol.

**Table 2:** Hazard ratios of all-cause and cardiovascular mortality stratified by interleukine-6 tertiles and continuous levels.

Variable	Tertile 1	Tertile 2	Tertile 3	Per SD increment
Interleukine-6 (pg/mL)				
Male	0.10–1.65	1.68–3.67	3.69–101.63	
Female	0.09–1.63	1.68–3.38	3.39–43.19	
No. of patients	238	241	239	

No. of all-cause deaths	2	9	36	
Person-years	545	595	523	
Rate per 1000 person-years	3.7	15.1	68.8	
Model 1*	[Reference]	**4.80 (1.03**–**22.42), *P* = 0.046**	**19.44 (4.38**–**86.29), *P* < 0.001**	**3.05 (2.23**–**4.18), *P* < 0.001**
Model 2^†^	[Reference]	**4.57 (0.95**–**21.93), *P* = 0.058**	**17.78 (3.93**–**77.65), *P* < 0.001**	**3.04 (2.22**–**4.17), *P* < 0.001**
Model 3^‡^	[Reference]	**4.06 (0.88**–**18.78), *P* = 0.073**	**17.12 (3.11**–**71.76), *P* < 0.001**	**2.93 (2.11**–**4.08), *P* < 0.001**

No. of CVD deaths	2	3	19	
Person-years	545	595	523	
Rate per 1000 person-years	3.7	5.0	36.3	
Model 1*	[Reference]	**1.32 (0.22**–**7.92), *P* = 0.760**	**10.69 (2.39**–**49.56), *P* < 0.001**	**2.74 (1.75**–**4.28), *P* < 0.001**
Model 2^†^	[Reference]	**1.22 (0.19**–**7.79), *P* = 0.831**	**9.65 (2.01**–**47.88), *P* < 0.001**	**2.34 (1.56**–**4.07), *P* < 0.001**
Model 3^‡^	[Reference]	**1.12 (0.16**–**7.77), *P* = 0.906**	**8.68 (1.88**–**37.51), *P* < 0.001**	**2.04 (1.34**–**3.68), *P* < 0.001**

The quartiles are stratified by gender and the interleukin-6 levels are log transformed. The values are HR (95% confidence interval).

*Adjusted for age, sex, and body mass index. ^†^Adjusted for the variables in model 1 plus smoking, alcohol use, hypertension, diabetes mellitus, low-density lipoprotein cholesterol, and high-density lipoprotein cholesterol. ^‡^Adjusted for the variables in model 2 plus C-reactive protein (log transformed).

**Table 3:** Hazard Ratios for All-cause and Cardiovascular Mortality According to Interleukine-6 Quartiles and Continuous Levels in Males.

Variable	Tertile 1	Tertile 2	Tertile 3	Per SD increment
Interleukine-6 (pg/mL)	**0.01–1.65**	**1.68–3.67**	**3.69–101.63**	
No. of patients	147	150	148	

No. of all-cause deaths	2	8	29	
Person-years	340	362	304	
Rate per 1000 person-years	5.9	22.1	95.4	
Model 1*	[Reference]	4.54 (0.92–22.41), *P* = 0.064	**16.34 (3.52**–**72.70), *P* < 0.001**	**3.18 (2.21**–**4.57), *P* < 0.001**
Model 2^†^	[Reference]	4.19 (0.88–20.02), *P* = 0.072	**13.89 (3.30**–**58.42), *P* < 0.001**	**3.16 (2.17**–**4.60), *P* < 0.001**
Model 3^‡^	[Reference]	3.55 (0.75–16.75), *P* = 0.109	**11.68 (3.04**–**37.56), *P* < 0.001**	**2.98 (2.12**–**4.18), *P* < 0.001**

No. of CVD deaths	2	3	18	
Person-years	340	362	304	
Rate per 1000 person-years	5.9	8.3	59.2	
Model 1*	[Reference]	1.32 (0.22–7.93), *P* = 0.759	**11.35 (2.49**–**51.78), *P* = 0.002**	**3.41 (2.05**–**5.67), *P* < 0.001**
Model 2^†^	[Reference]	1.14 (0.18–7.43), *P* = 0.892	**10.81 (2.05**–**57.13), *P* = 0.005**	**3.34 (1.99**–**5.62), *P* < 0.001**
Model 3^‡^	[Reference]	1.10 (0.16–7.74), *P* = 0.927	**8.37 (1.93**–**36.22), *P* = 0.005**	**3.10 (1.98**–**4.85), *P* < 0.001**

The interleukin-6 levels are log transformed. The values are HR (95% confidence interval). *Adjusted for age and body mass index.

^†^Adjusted for the variables in model 1 plus smoking, alcohol use, hypertension, diabetes mellitus, low-density lipoprotein cholesterol, and high-density lipoprotein cholesterol. ^‡^Adjusted for the variables in model 2 plus C-reactive protein (log transformed).
